# Choroidal Thickness in Eyes with Unilateral Ocular Ischemic Syndrome

**DOI:** 10.1155/2015/620372

**Published:** 2015-10-04

**Authors:** Dong Yoon Kim, Soo Geun Joe, Joo Yong Lee, June-Gone Kim, Sung Jae Yang

**Affiliations:** ^1^Department of Ophthalmology, College of Medicine, Chungbuk National University, Cheongju, Republic of Korea; ^2^Department of Ophthalmology, Gangneung Asan Hospital, College of Medicine, University of Ulsan, Gangneung 210-711, Republic of Korea; ^3^Department of Ophthalmology, Asan Medical Center, College of Medicine, University of Ulsan, Seoul, Republic of Korea

## Abstract

*Aim*. To analyze the subfoveal choroid thickness and choroidal volume in unilateral ocular ischemic syndrome (OIS). *Methods*. A retrospective review was conducted for all patients with unilateral OIS from October 2010 through June 2014. The subfoveal choroidal thickness (SFChT) and choroidal volume of both eyes were compared. *Results*. 19 unilateral OIS patients were included in this study. The mean SFChT of OIS eyes was significantly lower than that of fellow eyes (OIS eyes: 208.89 ± 82.62 *μ*m and fellow eyes: 265.31 ± 82.77 *μ*m, *P* < 0.001). The choroidal volume of OIS eyes was significantly smaller than that of fellow eyes (OIS eyes: 0.16 ± 0.05 mm^3^ and fellow eyes: 0.21 ± 0.05 mm^3^, *P* < 0.001). *Conclusion*. The choroidal thickness and volume of OIS eyes were smaller than those of unaffected fellow eyes. Decreased choroidal circulation caused by carotid artery stenosis might affect the discordance of choroidal thickness and choroidal volume.

## 1. Introduction

Ocular ischemic syndrome (OIS) is a potentially vision-threatening disorder caused by ocular hypoperfusion resulting from stenosis or occlusion of the common or internal carotid arteries [1]. OIS manifests as visual loss, orbital pain, and changes of the visual field. Chronic progressive ocular ischemia may lead to permanent blindness secondary to neovascular glaucoma and optic atrophy [2, 3].

Stenosis or occlusion of the common or internal carotid arteries affects not only retinal blood flow but also choroidal blood flow. Because the choroid is a vascular tissue layer encasing a cavernous space, certain characteristics of the choroid, particularly its thickness, have been challenging to study histologically. Recent studies reported that the subfoveal choroidal thickness can be measured noninvasively by enhanced depth imaging OCT (EDI-OCT) [4, 5]. The choroid plays an important role in the physiology of healthy eyes and in the pathogenesis of a variety of ocular diseases. Because the choroidal thickness changes with specific pathologies, measurements of choroidal thickness are becoming more common, and it is an accepted procedure in the clinic and in research [6–9].

A previous study reported 3 cases of OIS patients who showed lower choroidal thickness in affected eye [10]. We postulated that ocular hypoperfusion may affect choroidal thickness and tried to demonstrate a difference in choroidal thickness in ocular ischemic syndrome with a large number of patients. To our knowledge, subfoveal choroidal thickness has not been fully investigated in a large number of patients. Here, we compared the subfoveal choroid thickness and choroidal volume between eyes with OIS and healthy contralateral eyes in 19 patients.

## 2. Patients and Methods

### 2.1. Study Design and Participants

A retrospective review was conducted of all patients who were diagnosed with OIS at Asan Medical Center in Seoul, Korea, and Gangneung Asan Hospital in Gangneung, Korea, from October 2010 through June 2014. We diagnosed OIS in eyes with at least four of the following symptoms and signs: (1) visual loss, abrupt or gradual; (2) ocular pain; (3) iris rubeosis; (4) narrow retinal arteries; (5) dilated, nontortuous retinal veins; (6) midperipheral retinal hemorrhages; and (7) neovascularization of the optic disc in the posterior segment, in addition to two or more of the following fluorescein angiographic signs: (1) patchy, delayed choroidal filling; (2) increased retinal arteriovenous transit time; and (3) late retinal artery staining and definite stenosis of carotid artery in carotid doppler examination [11, 12]. Exclusion criteria included bilateral OIS eyes, presence of refractive error > ±4.0 diopters, choroidal neovascularization or other macular diseases that might affect vision, active intraocular inflammation and/or infection, or a history of any type of intraocular surgery (except cataract surgery), and diabetic retinopathy. We also excluded patients who underwent carotid endarterectomy before SD-OCT examination. The study was approved by the Institutional Review Board of Asan Medical Center and Gangneung Asan Hospital and this study followed the tenets of the Declaration of Helsinki.

### 2.2. Ophthalmic Examinations

All patients received a complete bilateral ophthalmic examination, including best corrected visual acuity (BCVA), by using the Snellen eye chart. All BCVA results were converted to the LogMAR scale. All patients also underwent refractive error assessment, biomicroscopic examination, fundus examination, fundus photography, FA (fluorescein angiography), and SD-OCT. A Heidelberg Spectralis device (Heidelberg Engineering, Heidelberg, Germany) was used to obtain SD-OCT images of the macula by applying a custom 25° × 25° volume acquisition protocol to obtain a set of high-speed scans from each eye. Using this protocol, 25 cross-sectional B-scan images were obtained, each of which was composed of 512 A-scans. Choroidal images were also obtained using the EDI technique.

**Figure 1:**
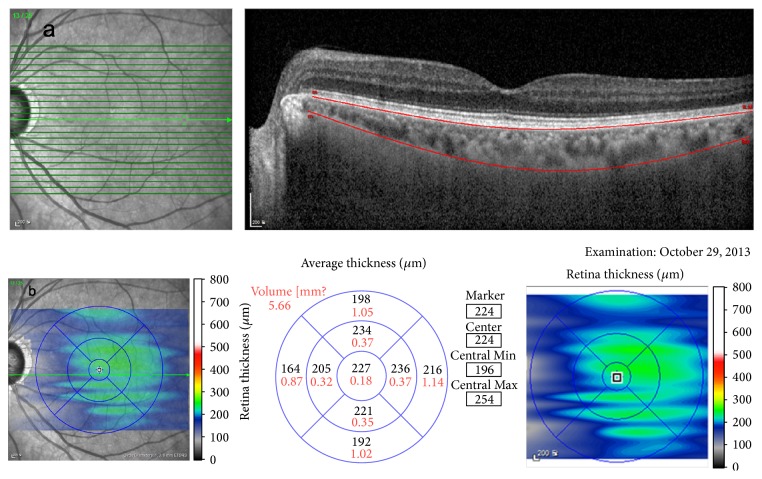
Choroidal volume measurement. For choroidal volume measurement, we manually moved the automatically segmented internal limiting membrane line to the retinal pigment epithelium and the automatically segmented retinal pigment epithelium lint to the chorioscleral junction. Once we changed the automatically segmented line, the choroidal volume was automatically calculated and displayed within the ETDRS grid.

SFChT was manually measured by two retina specialists (Dong Yoon Kim and Sung Jae Yang) using calipers and Heidelberg Eye Explorer software. For choroidal volume measurement, we manually moved the automatically segmented internal limiting membrane line to the retinal pigment epithelium and the automatically segmented retinal pigment epithelium line to the chorioscleral junction. Once we changed the automatically segmented line, the choroidal volume was automatically calculated and displayed within the ETDRS grid. We obtained five choroidal volume data points within 3000 *μ*m in the EDTRS grid (foveal center, superior, nasal, inferior, temporal) (Figure 1). Choroidal volume measurement was also conducted by two retina specialists (Dong Yoon Kim and Sung Jae Yang). From these data, we analyzed differences in SFChT and choroidal volume between the OIS eye and fellow eye.

### 2.3. Statistical Analysis

The independent *t*-test was used to compare OCT parameters between OIS eyes and fellow eyes. To assess the interrater reproducibility of the SFChT and choroidal volume measurements, a Bland-Altman plot was generated for each subject. SPSS version 21.0 (SPSS Inc., Chicago, IL) was used to perform all analyses, and *P* < 0.05 was considered statistically significant.

## 3. Results

19 patients were diagnosed with unilateral OIS at the Asan Medical Center and Gangneung Asan Hospital from October 2010 through June 2014.

Table 1 shows the clinical characteristics of unilateral ocular ischemic syndrome. The mean age of all included eyes was 68.79 ± 8.08 years. Among 19 patients, 15 and 4 patients were male and female, respectively. Among 19 OIS eyes, 13 and 6 eyes developed OIS in the right and left eye, respectively. The proportion of carotid stenosis of the OIS eye and fellow eye was 79.8% and 47.3%, respectively. The baseline LogMAR BCVA was significantly worse in OIS eyes than in the fellow eyes (OIS eye: 0.97 ± 0.75 and fellow eye: 0.16 ± 0.17, *P* < 0.001). The mean SFChT of OIS eyes was significantly lower than that of fellow eyes (OIS eyes: 208.89 ± 82.62 *μ*m and fellow eyes: 265.31 ± 82.77 *μ*m, *P* < 0.001). At foveal center, the choroidal volume of OIS eyes was significantly smaller than those of fellow eyes (Table 2, OIS eyes: 0.16 ± 0.05 mm^3^ and fellow eyes: 0.21 ± 0.05 mm^3^, *P* < 0.001), and the choroidal volume was also significantly smaller in OIS eyes than in the fellow eyes in the superior, nasal, inferior, and temporal quadrants within a 1000 *μ*m ETDRS grid.

**Figure 2:**
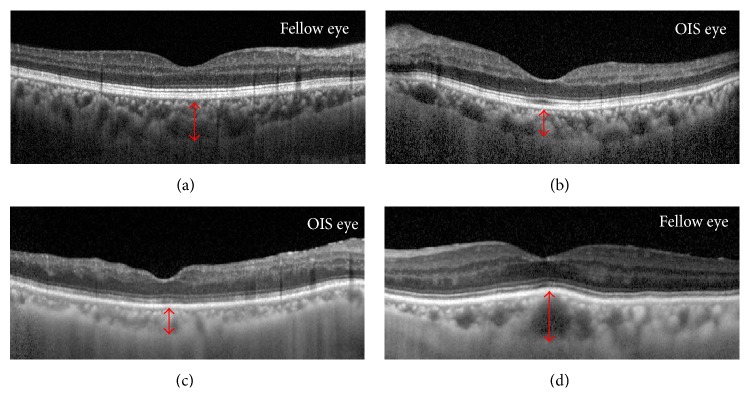
Optical coherent tomography (OCT) findings, choroidal volume, and fundus image of ocular ischemic syndrome (OIS) and fellow eyes. A 59-year-old man had unilateral OIS in the left eye (a, b). The visual acuity of his right and left eye was 1.0 and 0.4, respectively. The subfoveal choroidal thickness (SFChT) of the OIS eye (b) and fellow eye (a) was 204 *μ*m and 302 *μ*m, respectively. An 82-year-old man had unilateral OIS in the right eye (c, d). The visual acuity of his right and left eye was counting finger and 0.4, respectively. The subfoveal choroidal thickness (SFChT) of the OIS eye (c) and fellow eye (d) was 155 *μ*m and 279 *μ*m, respectively.

Two representative patients are described as follows. A 59-year-old man had unilateral OIS in the left eye. The visual acuity of his right and left eye was 1.0 and 0.4, respectively. On slit lamp examination, rubeosis at the iris and angle was found. SFChT and choroidal volume were larger in OIS eyes than in fellow eyes (Figures 2(a) and 2(b)). The patient underwent carotid endarterectomy because of severe carotid arterial stenosis. At 2 days after carotid endarterectomy, the intraocular pressure in the left eye was increased to the 30 mmHg. Because of the uncontrolled intraocular pressure, a drainage implant was inserted to control intraocular pressure. An 82-year-old man had unilateral OIS in the right eye. The visual acuity of his right and left eye was counting finger and 0.4, respectively. The SFChT and choroidal volume were larger in the fellow eye than in the fellow eye (Figures 2(c) and 2(d)).

**Figure 3:**
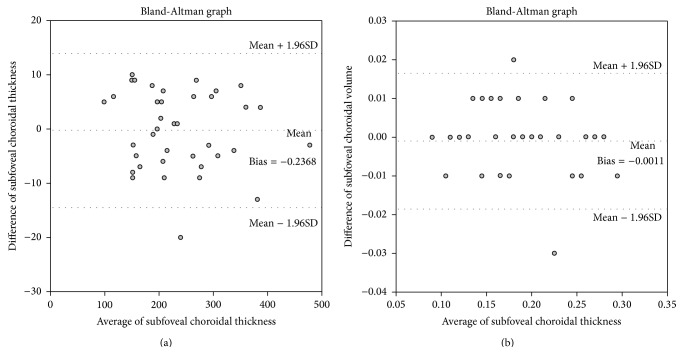
Bland-Altman plot of subfoveal choroidal thickness (SFChT) and choroidal volume. (a) Bland-Altman plot of SFChT measurements taken by two independent retina specialists. No substantial bias was found between the two sets of measurements. The 95% limit of agreement for the SFChT ranged from −14.40 to 13.93. (b) Bland-Altman plot of choroidal volume measurements showing no substantial bias between the two sets of measurements. The 95% limit of agreement for the choroidal volume measurement ranged from −0.186 to 0.165.

To evaluate interobserver reliability of SFChT and choroidal volume measure, Bland-Altman plots were generated and good agreement between the two sets of SFChT and choroidal volume measurements was observed (Figure 3).

## 4. Discussion

In this study, we compared the subfoveal choroid thickness and choroidal volume between eyes with OIS and healthy fellow eyes in 19 patients. Kang et al. reported that, compared with fellow eyes, the choroidal thickness of OIS eyes was significantly decreased [10]. In this study, we have demonstrated that unilateral OIS eyes have a thinner subfoveal choroid and lower choroidal volume than fellow eyes, indicating impaired choroidal circulation in patients with OIS.

Posterior segment changes, such as narrowed retinal arteries, dilated retinal veins, midperipheral retinal hemorrhages, microaneurysms, retinal neovascularization, and vitreous hemorrhage, are characteristic in OIS. These findings are associated with retinal ischemia and it is unclear how choroidal ischemia would affect the retinal ischemia and vice versa. It is also not certain whether the choroidal thickness change occurs earlier than retinal changes with the development of OIS. It is also not possible to know when the retina and choroid change occurs and whether choroidal thinning occurred before retinal changes or whether retinal changes occurred before choroidal thinning with the progression of OIS.

The BCVA was also significantly different between OIS eyes and fellow eyes. The baseline LogMAR BCVA was significantly worse in OIS eyes than in fellow eyes (OIS eye: 0.97 ± 0.75 and fellow eye: 0.16 ± 0.17, *P* < 0.001).

To our knowledge, subfoveal choroidal thickness and choroidal volume have not been fully investigated in a large number of patients. The strength of our current study is that it is the first to analyze choroidal volume and thickness difference in unilateral OIS eyes in a larger number of patients. However, our present study has limitations that are inherent to its retrospective design. The sample size of this study was also relatively small, which may have limited the statistical strength of the analysis. Therefore, future studies that examine a larger number of patients are needed to confirm the difference in choroidal thickness in unilateral OIS eyes, and it will be also necessary to observe how retina and choroid change with the progression of OIS by serial evaluation.

In conclusion, the choroidal thickness and volume of OIS eyes were smaller than those of unaffected fellow eyes. Decreased choroidal circulation caused by carotid artery stenosis may affect the discordance of choroidal thickness and choroidal volume.

## Figures and Tables

**Table 1:** Clinical characteristics of unilateral ocular ischemic syndrome.

Characteristic	OIS eyes	Fellow eyes	*P* value
Number of eyes	19		
Age (year)	68.79 ± 8.08		
Sex (male/female)	15/4		
Right/left	13/6		
Carotid stenosis (%)	79.8 (%)	47.3 (%)	<0.001^a^
Refractive error (SE)	0.46 ± 1.26	0.57 ± 1.20	0.689^a^
Baseline BCVA (LogMAR)	0.97 ± 0.75	0.16 ± 0.17	<0.001^a^
IOP (mm Hg)	19.47 ± 9.32	15.21 ± 3.24	0.082^a^

OIS: ocular ischemic syndrome; BCVA: best corrected visual acuity; SE: spherical equivalent; IOP: intraocular pressure.

^a^Independent *t*-test.

**Table 2:** Subfoveal choroidal thickness and choroidal volume in unilateral ocular ischemic syndrome.

	OIS eyes	Fellow eyes	*P* value^a^
Baseline SFChT (*μ*m)	208.89 ± 82.62	265.31 ± 82.77	<0.001
Choroidal volume			
Foveal center (mm^3^)	0.16 ± 0.05	0.21 ± 0.05	<0.001
Superior (mm^3^)	0.33 ± 0.10	0.40 ± 0.10	0.002
Nasal (mm^3^)	0.30 ± 0.11	0.37 ± 0.10	0.001
Inferior (mm^3^)	0.32 ± 0.11	0.39 ± 0.10	0.001
Temporal (mm^3^)	0.32 ± 0.09	0.41 ± 0.08	<0.001

OIS: ocular ischemic syndrome; SFChT: subfoveal choroidal thickness. ^a^Independent *t*-test.
